# Cavernous Hemangioma of the Tongue

**DOI:** 10.1155/2013/898692

**Published:** 2013-08-31

**Authors:** Krishna Kripal, Senthil Rajan, Beena Ropak, Ipsita Jayanti

**Affiliations:** ^1^Department of Periodontology, Rajarajeswari Dental College & Hospital, Bangalore, Karnataka 560060, India; ^2^Department of Oral and Maxillofacial Surgery, Rajarajeswari Dental College & Hospital, Bangalore, Karnataka 560060, India

## Abstract

Hemangioma is a benign tumor of dilated blood vessels. It is most commonly seen in the head and neck region and rarely in the oral cavity. Hemangiomas in the oral cavity are always of clinical importance and require appropriate treatment. We report here a case of a 34-year-old female patient with a swelling on the lateral surface of tongue which did not respond to the sclerosing agent and was finally confirmed as cavernous hemangioma on histological evaluation.

## 1. Introduction 

Hemangioma (Greek: Haima-blood; angeion vessel, omatumor) by definition can be defined as “a benign tumor of dilated blood vessels.” Hemangioma of head and neck appear a few weeks after birth and they grow rapidly. It is also known as port-wine stain, strawberry hemangioma, and Salmon patch. They are characterized by hyperplasia of blood vessels, usually veins and capillaries, in a focal area of submucosal connective tissue. It is almost never encapsulated. Whether this condition is a neoplastic or reactive state is uncertain; a reactive cause is favored.

Few of the reactive causes are, namely, hormonal changes, infections, and trauma. Clinically they may manifest as firm, pulsatile, warm masses and the venous malformations appear first in early childhood and clinically manifest as soft and easily compressible mass. According to the classification given by Mulliken and Glovacki in 1982, are divided the vascular deformities, into 2 groups: hemangiomas and the vascular malformations. The hemangiomas can also be classified depending on the vessel type involved or flow types such as the arterial and arteriovenous (high flow) type, capillary or venous (low flow) type [1].

This paper describes a case of a female patient who had a growth on her lateral border of the tongue which was diagnosed as cavernous hemangioma.

## 2. Case History

**Figure 1:**
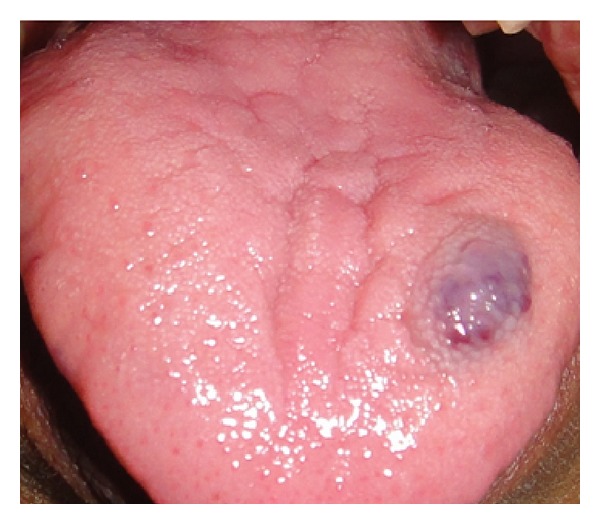
Preoperative photograph.

A 34-year-old female patient reported to the Department of Periodontology, Rajarajeswari Dental College and Hospital, Bangalore, India, with the chief complaint of swelling on the lateral surface of the tongue. The patient gave a history of trauma at the same site 10 years back. There was no history of associated pain or bleeding from the site. On general examination, the patient was normally built for her age with no defect in stature or gait. No relevant medical history was observed. On intraoral examination, there was a growth measuring about 1.5 cm × 1.5 cm, which was red in colour with a bluish hue present at the left lateral surface of the tongue which appeared to be sessile with no underlying attachment or relation with the muscles (Figure 1). The borders were well defined and there was no ulceration seen on the surface of the lesion. The growth was soft to palpate, and it showed blanching on application of pressure. A provisional diagnosis of capillary hemangioma was given based on the clinical findings.

## 3. Management

**Figure 2:**
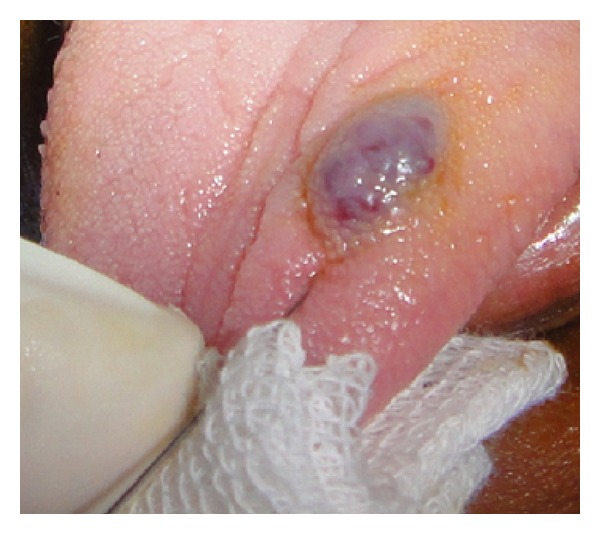
Administration of sclerosing agent.

**Figure 3:**
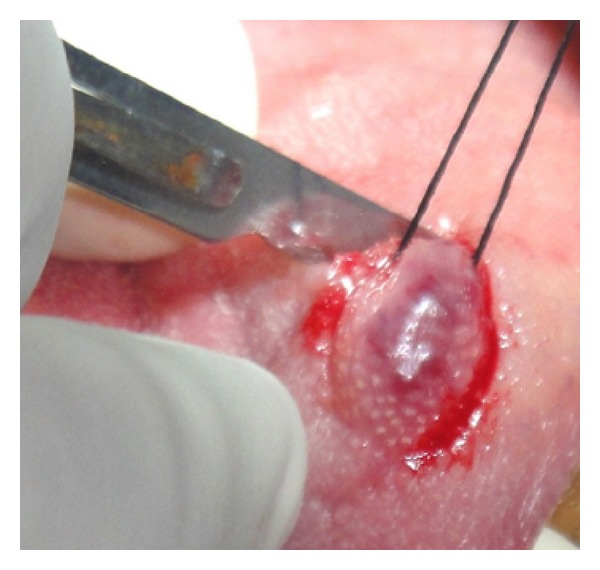
Surgical excision.

**Figure 4:**
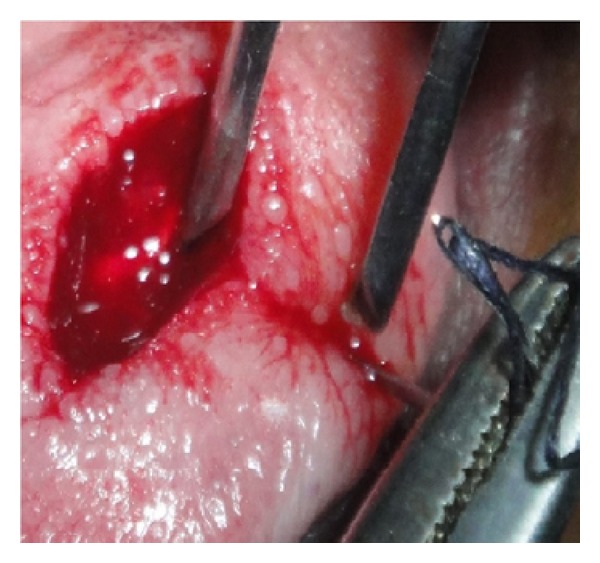
Sutures being placed.

**Figure 5:**
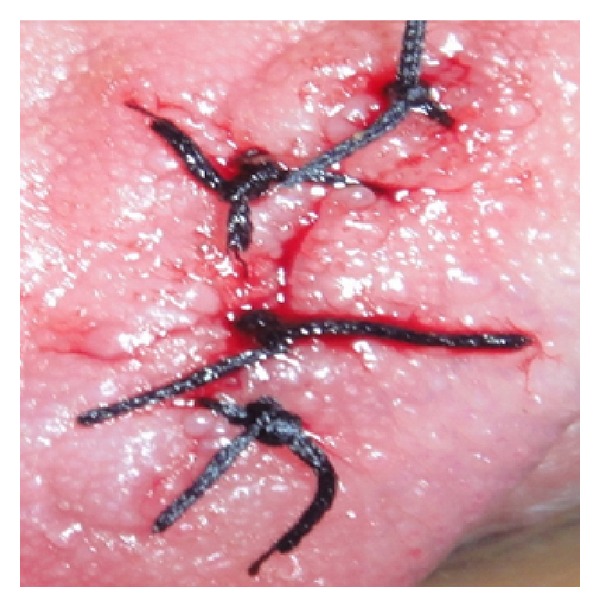
Interrupted sutures placed.

**Figure 6:**
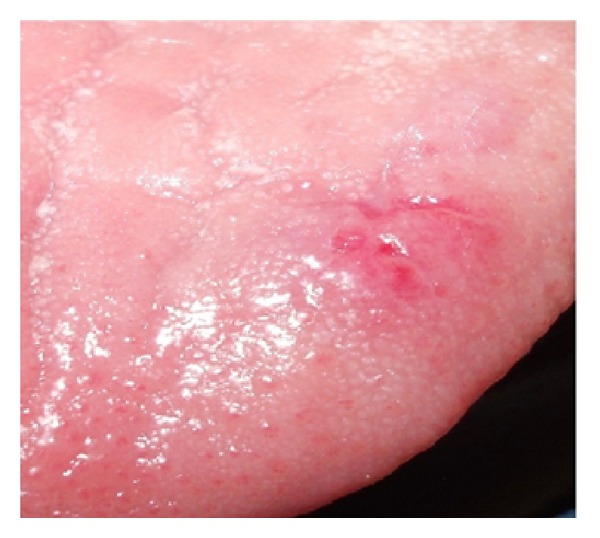
1 week postoperative photograph.

**Figure 7:**
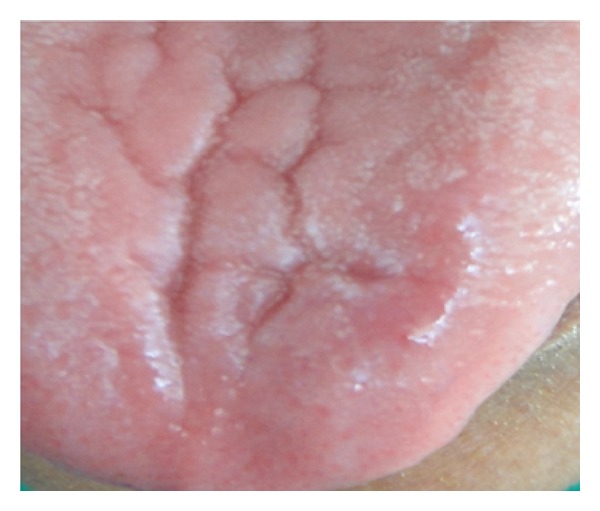
1 month postoperative photograph.

**Figure 8:**
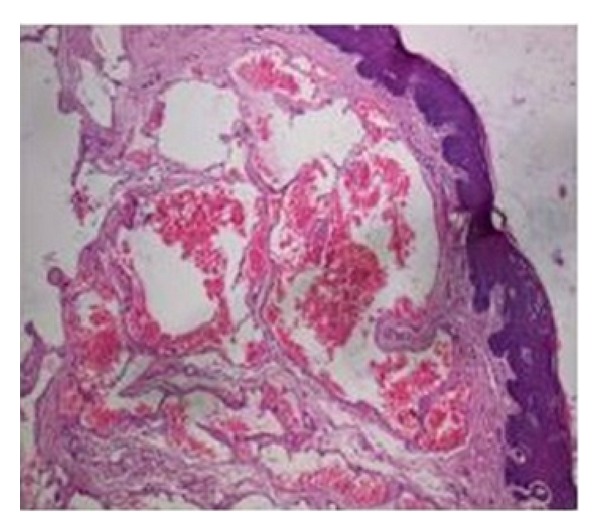
Histopathological picture.

 Initially a sclerosing agent was administered topically and the mass was observed for a period of 1 week (Figure 2). No change in the appearance and size of the mass was observed. After a period of 1 week, a surgical excision was carried out under local anesthesia. During the surgical procedure, a thread was tied around the base, and the lesion was stretched in an upward direction in order to get maximum accessibility (Figure 3). The mass was then excised out, and interrupted sutures were placed (Figures 4 and 5). During the surgical procedure, minimal amount of bleeding from the site was observed. The specimen was then sent for a histopathological examination. The healing was uneventful after a period of 1 week (Figure 6) and complete healing was seen after 1 month (Figure 7). The histopathology report confirmed the diagnosis of cavernous hemangioma with void capillary vessel (Figure 8). 

## 4. Discussion

Hemangiomas are the most common benign tumours of the head and neck in children, but their occurrence on the tongue is extremely rare. The tongue requires special consideration because of its susceptibility to minor trauma and consequent bleeding and ulceration, swallowing difficulties, and breathing problem, although the major concern is cosmetic in most cases. The hemangioma appears as soft mass, smooth or lobulated, and sessile or pedunculated and may vary in size from a few millimeters to several centimeters [2, 3]. They are usually deep red and may blanch on the application of pressure and if large in size, it might interfere with mastication [4, 5]. The superficial hemangiomas are often lobulated, and blanch under finger pressure and the deeper lesions tend to be dome-shaped with normal or blue surface coloration, and they seldom blanch. A lesion with a thrill or bruit or with an obviously warmer surface, is most likely a special vascular malformation, called arteriovenous hemangioma (arteriovenous aneurysm, A-V shunt, arteriovenous malformation), with direct flow of blood from the venous to the arterial system, bypassing the capillary beds.

 Various syndromes that are associated with the vascular malformation include the Osler-Weber-Rendu syndrome, Sturge-Weber syndrome, and blue rubber bleb nevus syndrome. A differential diagnosis of granuloma fasciale, insect bite, pyogenic granuloma, and angiosarcoma can be given for this condition.

 Clinical diagnosis was based on histopathological evaluation, which was confirmed to be a cavernous hemangioma. Histologically, the appearance of these lesions depends on the stage of the evolution. Early lesions may be very cellular with solid nests of plump endothelial cells and little vascular lumen. Established lesions comprise of well-developed, flattened, and endothelium-lined capillary channels of varying sizes in a lobular configuration. Involuting lesions show increased fibrosis and hyalinization of capillary walls with luminal occlusion [6]. In cavernous hemangioma, there is presence of large dilated blood sinuses with thin walls each showing an endothelial lining. The sinusoidal spaces are usually filled with blood although there might be presence of lymphatic vessels [7].

## 5. Conclusion

The appearance of cavernous hemangioma is a rare occurrence on the tongue. Early detection and biopsy are crucial in determining the clinical behavior of the tumour and potential complications. The treatment modality should be planned according to the diagnosis and prognosis of the particular vascular malformation.
